# The Synergistic Local Immunosuppressive Effects of Neural Stem Cells Expressing Indoleamine 2,3-Dioxygenase (IDO) in an Experimental Autoimmune Encephalomyelitis (EAE) Animal Model

**DOI:** 10.1371/journal.pone.0144298

**Published:** 2015-12-04

**Authors:** Young Eun Lee, Jaeyeol An, Kee-Hang Lee, Sung Su Kim, Hye Jin Song, Heejang Pyeon, Hyun Nam, Kyeongjin Kang, Kyeung Min Joo

**Affiliations:** 1 Department of Health Science and Technology, Samsung Advanced Institute for Health Sciences and Technology (SAIHST), Sungkyunkwan University, Seoul, Republic of Korea; 2 Department of Anatomy and Cell Biology, Sungkyunkwan University School of Medicine, Suwon, Republic of Korea; 3 Samsung Biomedical Research Institute, Samsung Medical Center, Seoul, Republic of Korea; 4 Department of Neurosurgery, Samsung Medical Center, Sungkyunkwan University School of Medicine, Seoul, Republic of Korea; University Hospital of Heidelberg, GERMANY

## Abstract

Neurodegenerative diseases provoke robust immunological reactions in the central nervous system (CNS), which further deteriorate the neural tissue damage. We hypothesized that the expression levels of indoleamine 2,3-dioxygenase (IDO), an enzyme that has potent immune suppressive activities, in neural stem cells (NSCs) would have synergistic therapeutic effects against neurodegenerative diseases, since NSCs themselves have low IDO expression. In this study, the synergistic immune suppressive effects of rat fetal NSCs expressing IDO (rfNSCs-IDO) were validated by mixed leukocyte reaction (MLR) *in vitro* and an experimental autoimmune encephalomyelitis (EAE) animal model *in vivo*. rfNSCs-IDO showed significantly more suppressive effects on T cell proliferation in the MLR compared to control rfNSCs (rfNSCs-Cont). Importantly, IDO inhibition using 1-methyl-DL-tryptophan (1-MT), an IDO inhibitor, reversed the synergistic effects, confirming IDO-specific effects in rfNSCs-IDO. In the EAE animal model, systemic rfNSCs-IDO injections resulted in significant local immune suppression in the cervical lymph nodes and CNS, evidenced by a reduction in the number of activated T lymphocytes and an increase in regulatory T cell numbers, which induced significantly fewer clinical symptoms and faster recovery. In contrast, rfNSCs-Cont failed to reduce symptoms in the EAE animal models, although they showed local immune suppression, which was significantly less than that in rfNSCs-IDO. Taken together, IDO expression in NSCs synergistically potentiates the immune suppression activities of NSCs and could be applicable for the development of therapeutic modalities against various neurodegenerative diseases.

## Introduction

The central nervous system (CNS) has a limited ability to regenerate. Therefore, the onset and progression of neurodegenerative diseases can result in permanent impairment of brain functions. Therapeutic interventions using neural stem cells (NSCs) have been reported for various neurodegenerative diseases, including multiple sclerosis, traumatic brain injury, stroke, Parkinson’s disease, Huntington’s disease, Alzheimer’s disease, spinal cord injury, and epilepsy [1–8].

NSCs have a potential to differentiate into neurons and neuroglia and can be obtained from both fetal and adult brains [9,10]. Moreover, the clinical application of NSCs is reported to be effective against neurodegenerative diseases without causing serious immune complications [11,12]. However, in spite of the benefits of NSCs, the underlying therapeutic mechanism of NSCs still needs to be elucidated. One of the possible therapeutic mechanisms of NSCs is a bystander effect that exerts restoration in the injured brain in a cell-autonomous manner [13,14]. The bystander effect of NSCs is partly mediated by the anti-inflammatory effect and secretion of neurotrophic factors [15,16]. Due to a limitation in newly differentiated neural cells’ ability to immediately repair impaired complex neuronal functions, the bystander effects are considered to be crucial factors for the NSC therapy.

Normally, the CNS is protected from lymphocyte infiltration by the blood-brain-barrier (BBB). However, under neuropathological conditions, the BBB can be damaged and even broken, leading to a massive infiltration of lymphocytes into the CNS. Moreover, neuropathological conditions become more aggressive due to the boosted inflammation within the CNS [17,18]. Therefore, while the primary damage of the CNS is derived from the onset of disease, the secondary damage during the late phase of disease depends on inflammation, and it seems to be fatal [19,20]. It has been reported that microglia and lymphocytes play important roles in the pathological progression and are involved not only in the acute neurodegenerative diseases, including stroke and spinal cord injury, but also in the chronic diseases, such as Alzheimer’s disease [21]. Due to current limitations in the modulation of primary damage to the CNS, the prevention of secondary damage by inflammation seems to be a promising therapy.

Indoleamine 2, 3-dioxygenase (IDO) is a tryptophan-metabolizing enzyme that was found to be expressed by dendritic cells [22]. IDO metabolizes tryptophan to produce metabolites, kynurenines, which recruits regulatory T cells, modulating inflammation. A previous report documented the strong immune regulatory effect of IDO in chronic inflammatory disease [23]. In this study, we expected to see a synergic immunosuppressive effect of NSCs expressing IDO in the neuropathological condition, since the NSCs do not normally express high levels of IDO. To validate the immune modulatory effects of NSCs expressing IDO, we carried out a mixed leukocyte reaction (MLR) *in vitro* and transplanted NSCs expressing IDO into an experimental autoimmune encephalomyelitis (EAE) animal model, which mimics human multiple sclerosis (MS) [24].

## Materials and Methods

### Cell culture

Primary rat fetal NSCs (rfNSCs) were purchased (Life Technologies, Carlsbad, CA, USA) and grown in complete medium consisting of KnockOut DMEM/F-12 (Life Technologies) supplemented with StemPro NSC SFM supplement (Life Technologies), 20 ng/mL recombinant human EGF (R&D systems, McKinley, MN, USA), 20 ng/mL recombinant human basic FGF (R&D Systems) and penicillin/streptomycin (P/S) (Life Technologies). For adherent culture, cells were plated at a density of 5 × 10^5^ with complete medium in the 20 μg/mL poly-L-ornithine (PLO)- (Sigma-Aldrich, St. Louis, MO, USA) coated T25 flask (BD Biosciences Pharmingen, Heidelberg, Germany) and incubated for 4 days in a humidified 5% CO_2_ atmosphere at 37°C. 293FT cells were purchased (American Type Culture Collection, Manassas, VA, USA) and cultured in DMEM (Life Technologies) containing 10% FBS (Life Technologies), 1% P/S, 1% L-Glutamine (Life Technologies), 1% MEM Non-Essential Amino Acid Solution (MEM NEAA; Sigma-Aldrich) in a humidified atmosphere of 5% CO_2_ at 37°C.

### Immunocytochemistry

rfNSCs were fixed with 4% paraformaldehyde (Biosesang, Sungnam, Korea) for 15 minutes (mins), washed three times with 0.1% PBST (0.1% Triton X-100 in PBS), and incubated with primary antibodies at 4°C overnight. Primary antibodies were diluted in 0.1% bovine serum albumin (Sigma), 10% normal horse serum (Vector laboratories, Burlingame, CA, USA), and 0.3% Triton-X 100 in PBS at the following working concentrations: Nestin (1:200, Neuromics, Edina, MN), NeuN (1:200, Millipore, Billerica, MA), Olig2 (1:500, Millipore), GFAP (1:200, Sigma-Aldrich). After incubation with primary antibodies, a secondary antibody, Alexa Fluor 594 (1:500, Life Technologies) was applied to cells for 1 hour (hr) at room temperature in the dark. Cellular nuclei were counterstained with DAPI (1:1000, Sigma-Aldrich) for 5 mins. Slides were observed using a confocal laser scanning microscope (Fluoview FV 300, Olympus, Japan).

### Western Blotting

Cells were lysed in the RIPA lysis buffer consisting of 15 mM NaCl, 1% NP-40, 0.5% sodium deoxycholate, 0.1% SDS, and 50 mM Tris (pH 8.0). After centrifugation at 10,000 g for 5 mins, the supernatant was harvested. The concentration of protein was determined by a BCA protein assay kit (Life Technologies). 20 μg protein was separated on SDS-polyacrylamide gel electrophoresis for 2 hours (hrs) at 100 V, transferred onto a nitrocellulose membrane (GE Healthcare, Little Chalfont, United Kingdom) for 1 hr at 100 V, and then probed with an anti-actin (1:500, Santa Cruz Biotech, Santa Cruz, CA, USA) or IDO (1:500, Santa Cruz Biotech) antibody. The primary antibodies were then incubated with goat HRP-conjugated anti-mouse (1:100, Life Technologies) or anti-rabbit IgG antibody (1:100, Life Technologies) against actin and IDO, respectively. The antibodies were visualized by the Super ECL solution (GE Healthcare) following the manufacturer’s instructions.

### RT-PCR

The total RNA of rfNSCs was isolated using an RNeasy Plus Mini kit (Qiagen, Hilden, Germany) following the manufacturer’s recommendations. cDNA was synthesized from 1 μg of total RNA using a first-strand cDNA synthesis kit (Life Technologies) following the manufacturer’s instructions. PCR was conducted with 1 μL of first-strand cDNA product and iPfu DNA polymerase (Intron Biotechnology, Sungnam, Korea) with 35 amplifications using specific primers for GAPDH (forward primer: 5’-TGAAGGTCGGAGTCAACGGATTTGTG, reverse primer: 5’-CATGTGGGCCATGAGGTCCACCAC), IDO (forward primer: 5’-CACCATGCCTCACAGTCAAATATCTCCT, reverse primer: 5’-CTAAGGCCAACTCAGAAGGGCTTTCTT).

### Construction of Lentiviral Vectors and Establishment of Genetically Modified rfNSCs

A cloning package including an entry (pENTR/D-TOPO) and lentiviral destination (pLenti7.3/V5-DEST) vector, was used (Life Technologies) to construct an IDO-expressing lentiviral vector [25]. The PCR product of the IDO gene was delivered to the entry vector using the TOPO reaction and then transferred to the lentiviral destination vector through LR recombination following the manufacturer’s instructions using Gateway Cloning Technology (Life Technologies). The packaged lentivirus was produced in 293FT cells using the lentivirus destination vector, helper vectors [VSVG and PAX2 (Life Technologies)], and a Calcium Phosphate (CaPO_4_) Transfection Kit following the manufacturer’s protocol (Life Technologies). The supernatant was collected twice; after 48 and 72 hrs of transfection. The viral titer was determined by transduction of rfNSCs with serial dilutions of the viral supernatant and quantified by fluorescent microscope and flow cytometry to calculate multiplicity of infection (MOI) value. rfNSCs (1 × 10^5^ cells/well in a 6-well plate) were transduced with control or IDO expressing lentivirus with MOI = 1 and 6 μg/mL polybrene (Sigma-Aldrich) at passage 2. Control cells were generated using the pLenti7.3/V5-DEST vector with EmGFP gene under PGK promoter. Transduced rfNSCs were cultured in the complete medium. The transduction efficiency was examined by EmGFP-positive cell ratio in FACS analysis 48 hrs after lentiviral infection. The transduction efficacy was more than 80%. The average transduction unit (TDU) was 1.6 × 10^7^/mL. After transduction, the stable maintenance of EmGFP expression was observed by fluorescent microscope and flow cytometry up to 5 *in vitro* passages.

### Rat T cell isolation

Rat splenocytes were enzymatically and mechanically dissociated from 6-week-old SD rat spleens. Collected cells were labeled with rat anti-T cell microbeads (OX52, Miltenyi Biotech, Bergisch Gladbach, Germany) and loaded onto a magnetic associated cell sorting (MACS) LS column (Miltenyi Biotech) following the manufacturer’s protocol. The positive fraction of the loaded cells was collected and used for further experiments.

### *In vitro* T cell proliferation assay

8 × 10^3^ rfNSCs expressing either EmGFP alone (control), or IDO and EmGFP were seeded on PLO pre-coated 96-well plates in the complete media. After 2 days, purified rat T lymphocytes (2 × 10^5^, rfNSCs:T cells = 1:25) were added on the same plates and co-cultured in RPMI1640 medium containing 10% FBS, 1% P/S, 1% L-Glutamine, 1% MEM NEAA, and 1% 2-Mercaptoethanol (Sigma-Aldrich). To activate T cell proliferation, 10 μg/mL of Concanavalin A (ConA, Sigma-Aldrich) was applied to the co-cultured cells and maintained for 48 hrs. ^3^H-Thymidine (40 μCi/mL) was administered for 17 hrs before the quantitation of radioactivity using a beta counter. For the IDO inhibition assay, 0.5 mM of 1-methyl-DL-tryptophan (1-MT, Sigma-Aldrich), an IDO inhibitor, was applied to the NSC medium when NSCs were seeded on the 96-well plate.

### Experimental autoimmune encephalomyelitis (EAE) animal model

All animal experiments were approved by the appropriate Institutional Review Boards of the Seoul National University College of Medicine (Seoul, Korea; SNUIBC-R111205-1) and conducted in accordance with the ‘National Institute of Health Guide for the Care and Use of Laboratory Animals’ (NIH publication No. 80–23, revised in 1996). Female 6-week-old SD rats (130 g) were anesthetized by intraperitoneal injection of 10 mg/kg Ketamine (Yuhan, Seoul, Korea) and 65 mg/kg Xilazine (Bayer, Leverkusen, Germany) before all surgery to minimize suffering. They immunized intradermally with 50 μg of rat myelin oligodendrocyte glycoprotein (MOG; Sigma-Aldrich) in 0.2 mL of saline and 0.2 mL complete Freund’s adjuvant (CFA; Sigma-Aldrich) consisting of 1 mg/mL inactivated *Mycobacterium tuberculosis*. Animals were assessed daily according to the six-graded clinical scores of EAE; no clinical signs (grade 0), partial loss of tail tonicity (grade 0.5), complete loss of tail tonicity (grade 1), flaccid tail and abnormal gait (grade 2), hind leg paralysis (grade 3), hind leg paralysis with hind body paralysis (grade 4), hind and foreleg paralysis (grade 5), and death (grade 6). About 75% of the animals developed a monophasic EAE. 1 × 10^6^ control rfNSCs or rfNSCs expressing IDO (passage 3) in 200 μL HBSS were injected into the tail vein (n = 14 for each group) at 5 days post immunization (dpi). Alternatively, the same volume of HBSS was injected for the control group. Samplings for analysis were performed at 12 dpi when animals exhibited clinical symptoms of the EAE. All animals were checked for their health during the entire experimental period at least once a day after surgery. For animals who experienced paralysis, food and water were positioned lower to reach easily. Rats were euthanized in a CO_2_ chamber when they showed 20–30% reduction of body weight or were moribund.

### Flow cytometry analysis

Dissociated cells from spleens and cervical lymph nodes were analyzed by flow cytometry (FACSCalibur; BD Biosciences Pharmingen). Fluorescein isothiocyanate (FITC)-conjugated mouse monoclonal antibody against rat CD3 (1:1000, BD Biosciences Pharmingen), phycoerythrin (PE)-conjugated mouse monoclonal antibodies against rat CD45R (1:500, BD Biosciences Pharmingen), or rat CD25 (1:500, BD Biosciences Pharmingen). Intracellular protein FoxP3 was stained with an allophycocyanin (APC)-conjugated mouse monoclonal antibody using a FoxP3/Transcription Factor Staining Buffer Set (00-5523-00, eBioscience, San Diego, CA, USA) according to manufacturer’s protocol.

### Immunohistochemistry (IHC)

Rat brain tissues were embedded in OCT compound (Lab-Tek, Ames, Iowa, USA), and sectioned into 7-μm-thick sections. Infiltration of lymphocytes was assessed by hematoxylin and eosin (H&E) staining in the cerebral cortex (n = 7 for each group). Migration of injected rfNSCs in the brain was detected by immunofluorescent staining using anti-EmGFP antibody (Life Technologies). Xenograft tumors derived from SN12C-GFP renal carcinoma cells which express EmGFP stably (Metabio, Seoul, Korea) was used as positive control. For xenograft tumors, 1 × 10^6^ SN12C-GFP cells were transplanted into the subcutaneous tissue of BALB/c-nu mice. Average depth of lymphocyte infiltration was calculated for each animal and then compared. Various types of lymphocytes were stained with following antibodies: mouse anti-rat CD3 antibody (1:100, BD Biosciences Pharmingen) for T lymphocytes, mouse anti-rat CD25 antibody (1:100, BD Biosciences Pharmingen) for activated T lymphocytes, and anti-mouse/rat FoxP3 antibody (1:100, eBioscience) for regulatory T lymphocytes. Alexa Fluor 488 green (1:500, Life Technologies) was used as a secondary antibody. Visualized lymphocytes were analyzed in the cerebral cortex (n = 7). Average numbers of visualized lymphocytes were calculated for each animal and then compared.

### Statistical analysis

Statistical analysis was performed using the Student’s t-test. For EAE statistics, Mann-Whitney U test was used. Data were analyzed using GraphPad Prism 5 software. All tests were two tailed and values were calculated as the means ± standard deviation (SD) or were expressed as a percentage of controls ± SD. P value less than 0.05 was considered statistically significant.

## Results

### Rat fetal NSCs expressing IDO

**Fig 1:**
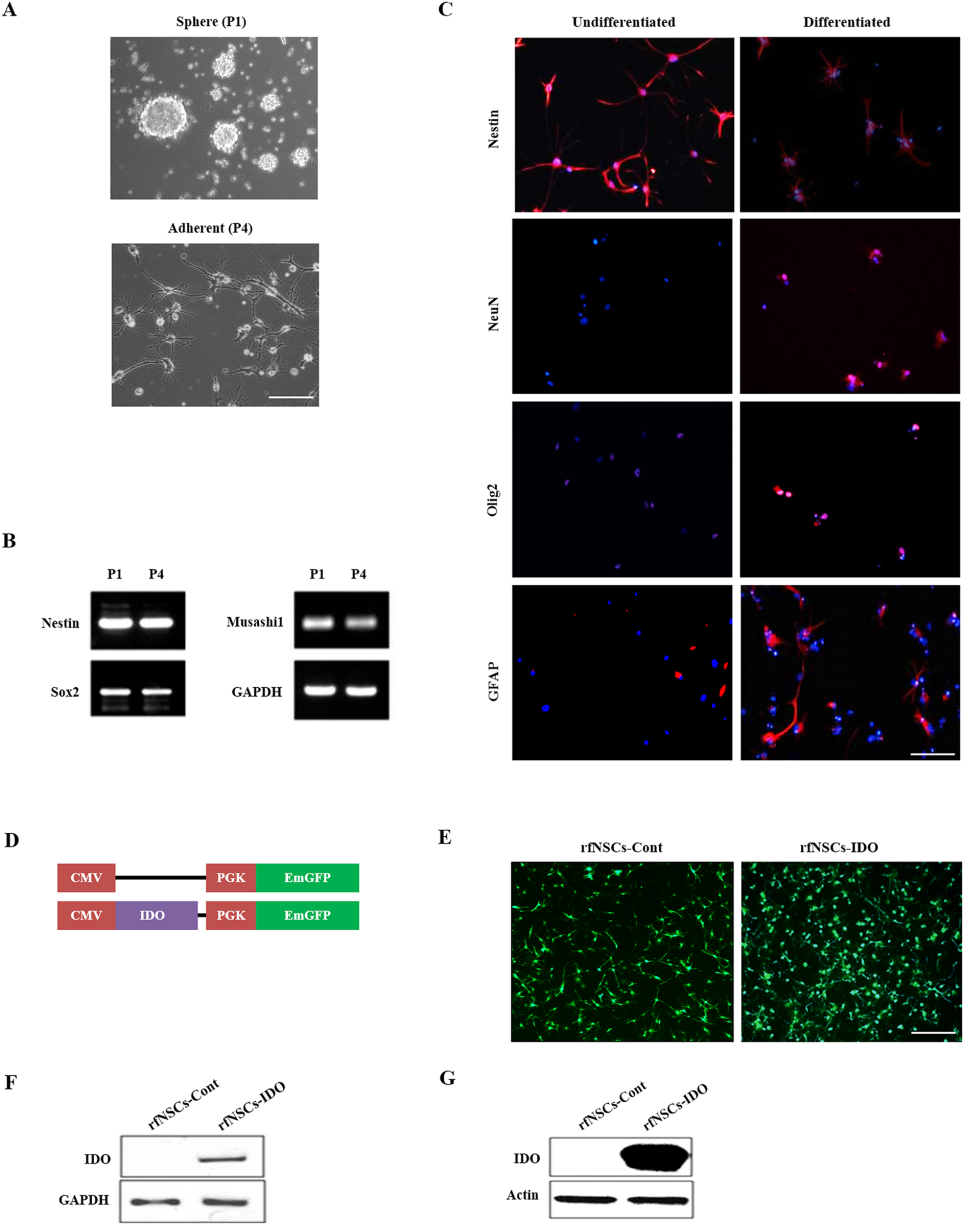
Characterization of rfNSCs and generation of IDO expressing NSCs. rfNSCs were cultured in two different methods *in vitro*. (A) rfNSCs initially formed neurospheres under suspension culture conditions and were then maintained adherently on PLO-coated plates. Scale bars, 50 μm. (B) The expression of NSC markers (Nestin, Sox2, and Musashi1) was confirmed by RT-PCR. As shown, there was no difference in the expression of NSC markers in both culture methods. (C) Undifferentiated rfNSCs were positive for Nestin. However, differentiated rfNSCs showed an increased expression of neural cell markers such as Olig2 (oligodendrocyte), GFAP (astrocyte), and NeuN (neuron). Scale bars, 50 μm. (D) Control and IDO expressing vector map scheme. IDO and EmGFP genes were inserted into the lentiviral vector, which were expressed by dual promoters, the CMV promoter and the PGK promoter, respectively. (E) After infection, the expression of EmGFP as a selection marker was observed under fluorescent microscope. rfNSCs-Cont (left) and rfNSCs-IDO (right) expressed EmGFP. Scale bars, 100 μm. The expression level of IDO was confirmed by (F) RT-PCR and (G) western blot.

Rat fetal NSCs (rfNSCs) were cultured in KnockOut DMEM/F12 medium containing 20 ng/mL EGF and 20 ng/mL bFGF forming neurospheres (Fig 1A). After several passages, rfNSCs were adhered on the plate coated with PLO in the same culture conditions (Fig 1A). To confirm the stem cell characteristics of rfNSCs, the expression of NSC markers such as Nestin, Sox2, and Musashi1 was compared by RT-PCR (Fig 1B) between the neurosphere culture at P1 and adherent culture at P4. The results showed that the expression of NSC markers was similar between both culture methods which is consistent with our prior research [26]. Differentiation was carried out in the complete medium without EGF and bFGF for 2 weeks, which resulted in the decreased expression of NSC markers such as Nestin and increased expression of differentiated neural cell markers such as NeuN (neuron), Olig2 (oligodendrocyte), and GFAP (astrocyte) (Fig 1C).

We engineered rfNSCs to express IDO since they show very low endogenous levels of IDO [27]. A lentiviral vector was designed to express IDO and EmGFP controlled by the CMV and PGK promoters, respectively (Fig 1D). As a control, we used a vector which expressed EmGFP alone (Fig 1D). We transduced control or IDO expressing lentivirus to rfNSCs (rfNSCs-Cont and rfNSCs-IDO, respectively). As designed, the rfNSCs-Cont and rfNSCs-IDO highly expressed EmGFP (Fig 1E). After infection, the stable expression of IDO in rfNSCs-IDO was confirmed by RT-PCR (Fig 1F) and western blot assay (Fig 1G) at passage 3, while rfNSCs-Cont showed no IDO signal. rfNSCs-Cont were used as control cells in following experiments.

### *In vitro* anti-inflammatory effects of rfNSCs expressing IDO

**Fig 2:**
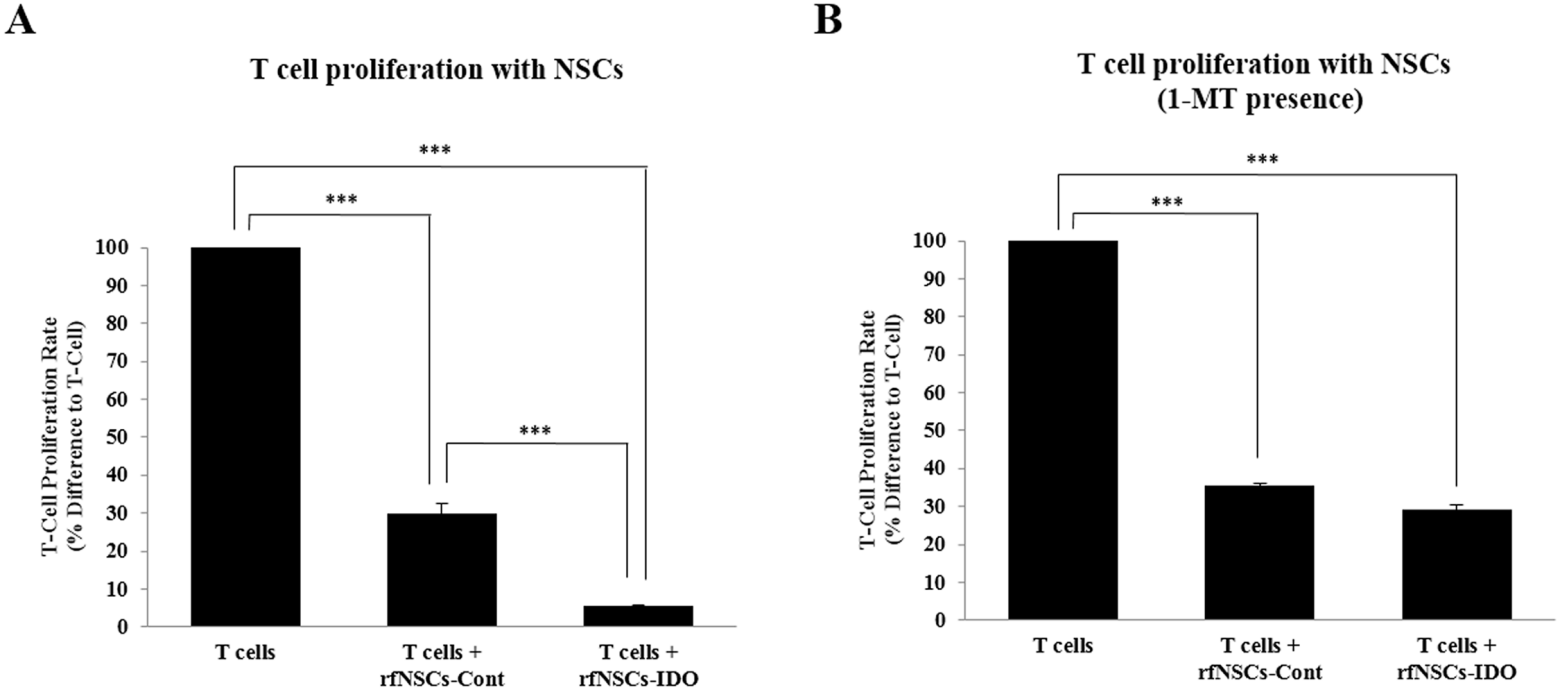
Suppression of T cell proliferation by rfNSCs expressing IDO. (A) The result of mixed leukocyte reaction showed that T cell proliferation was significantly suppressed by both rfNSCs-Cont and rfNSCs-IDO groups. Moreover, the T cell number in the rfNSCs-IDO group was significantly reduced compared to that in the rfNSCs-Cont group. (B) With the treatment of IDO inhibitor, 1-MT (1-methyl-DL-tryptophan) at a 0.5 mM concentration, the T cell number in the rfNSCs-IDO group became similar to that in the rfNSCs-Cont group. (n = 3, *** p < 0.001).

We analyzed the anti-inflammatory effect of rfNSCs on T lymphocyte proliferation *in vitro*. rfNSCs-Cont or rfNSCs-IDO were co-cultured with T lymphocytes which were isolated from rat spleen using anti-CD3 antibody (NSCs:T lymphocytes = 1:25). When the T lymphocytes were activated with ConA (10 μg/mL), NSCs alone were sufficient to inhibit T lymphocyte proliferation by 1/4 to 1/5 fold (Fig 2A). This is well-correlated with the previous reports showing that NSCs have inhibitory effect on T lymphocyte proliferation *in vitro* [28]. However, rfNSCs-IDO significantly suppressed T lymphocyte proliferation compared to rfNSCs-Cont (Fig 2A). The synergic suppression mediated by rfNSCs-IDO was observed as a 5-fold decrease of T cell proliferation compared to rfNSCs-Cont. To confirm the synergistic immune suppression was mediated by IDO, 1-MT, a specific IDO inhibitor was administered to rfNSCs-IDO. In the presence of 0.5 mM 1-MT, the synergic suppression of T lymphocyte proliferation by rfNSCs-IDO was decreased a level similar to that of to rfNSCs-Cont (Fig 2B). We therefore confirmed the immune suppressive effect of IDO was completely restored to the control level by 1-MT.

### Anti-inflammatory effect of rfNSCs expressing IDO *in vivo*

Next, we tested the anti-inflammatory effect of rfNSCs-IDO using the experimental autoimmune encephalomyelitis (EAE) animal model. EAE is an experimentally-induced autoimmune disease of the CNS mediated by activated T lymphocytes that represents an animal model of MS, a human demyelinating disease [29]. Notably, EAE is characterized by the presence of abnormally infiltrating T lymphocytes and macrophages into the broad area of the brain with a broken blood-brain-barrier (BBB) and the activation of microglia and astrocytes during the acute stage of EAE [30,31].

**Fig 3:**
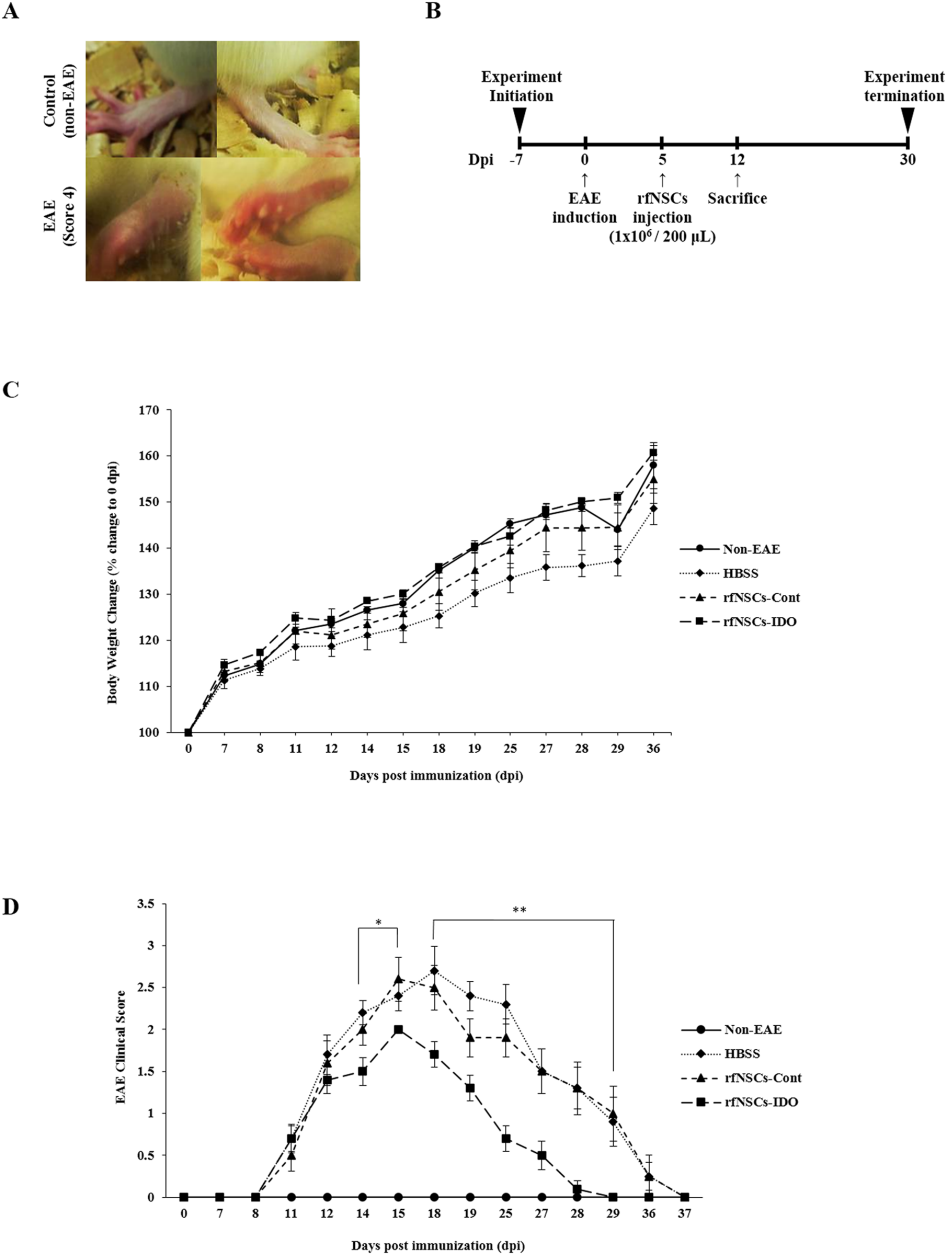
Immunosuppressive effect of rfNSCs-IDO in EAE animal model. Immunization with MOG and CFA induced acute inflammation in 6 week-old female SD rats. (A) EAE-induced rats exhibited swollen feet with hind limb paralysis. (B) The intravenous injection of rfNSCs-Cont, rfNSCs-IDO, or HBSS was performed through the rat tail vein at 5 dpi after EAE induction. The total EAE experiment took 37 days. (C) The rfNSCs-IDO group maintained similar body weight to non-EAE control group, whereas the HBSS group showed weight loss throughout the experimental period. (D) According to EAE clinical scoring chart, it was observed that the recovery rate of rfNSCs-IDO group was significantly faster than that of HBSS control group (n = 7 for each group, * *p* < 0.05, ** *p* < 0.01).

We injected the mixture of myelin oligodendrocyte protein (MOG) and complete Freud’s adjuvant (CFA) intradermally on the hind foot pads of animals to induce the acute inflammation. To evaluate the stages of EAE induction, we blindly observed the neurological symptoms of animals according to 6 graded parameter chart from the work of Einstein and colleagues [32]. At 7 days post immunization (dpi), immunized rats started to exhibit clinical symptoms of EAE including the loss of tail tonicity, abnormal gait, and continuous progression of disease. At around 15 to 18 dpi, animals showed scoring up to grade 4 and exhibited features such as hind leg paralysis and hind body paralysis with physical symptoms of reddish and swollen hind feet [33] (Fig 3A).

1 × 10^6^ rfNSCs-Cont or rfNSCs-IDO in 200 μL HBSS were injected into the tail vein at 5 dpi. Alternatively, the same volume of HBSS was injected for the control group (n = 14 for each group). Samplings including spleen, lymph node, and brain were performed at 12 dpi when animals exhibited clinical symptoms of the EAE (n = 7 for each group). The remaining animals (n = 7 for each group) were maintained to access the EAE clinical score and body weight up to 36 dpi, and then the experiments were terminated (Fig 3B).

Rats in all group showed a tendency toward increased body weight throughout the experimental period, but EAE-induced animals had relatively lower body weights compared to the normal non-EAE group. Although it was not statistically significant, the average body weight of the rfNSCs-IDO group was maintained at a level more similar to the non-EAE group compared to the rfNSCs-Cont group (Fig 3C). In the EAE control group, the clinical score of EAE was highest at 18 dpi with average score of grade 4 and it took more than 20 days to recover. Similarly, the rfNSCs-Cont group showed a highest clinical score at 15 dpi and it also took more than 20 days to become a normal clinical score. Conversely, the rfNSCs-IDO group was scored as grade 2, significantly lower than the other two testing groups, and showed a maximum score at 15 dpi. It took only 14 days to recover (Fig 3D).

Taken together, the rfNSCs-IDO group showed significantly lower clinical scorings with less body weight loss. Since higher clinical scorings with weight loss could reflect the progression of disease, our results indicated that the injection of rfNSCs-IDO synergistically minimized the acute inflammatory response in the EAE animal model. This could highlight the efficient immunosuppressive effect of rfNSCs-IDO in neural inflammatory condition *in vivo*.

### Immunosuppressive mechanisms of rfNSCs expressing IDO

To analyze the immunosuppressive mechanisms of rfNSCs-IDO in the EAE animal model, spleens and cervical lymph nodes were collected from all experimental animals (n = 7 for each group). The purpose of selecting spleen and cervical lymph node as test samples was to validate the inflammatory responses by quantifying lymphocytes in the systemic and regional aspect, respectively. Isolated lymphocytes were stained with antibodies against T lymphocytes (CD3^+^), B lymphocytes (CD45R^+^), activated T lymphocytes (CD3^+^ and CD25^+^), and regulatory T lymphocytes (CD3^+^, CD25^+^, and FoxP3^+^).

**Fig 4:**
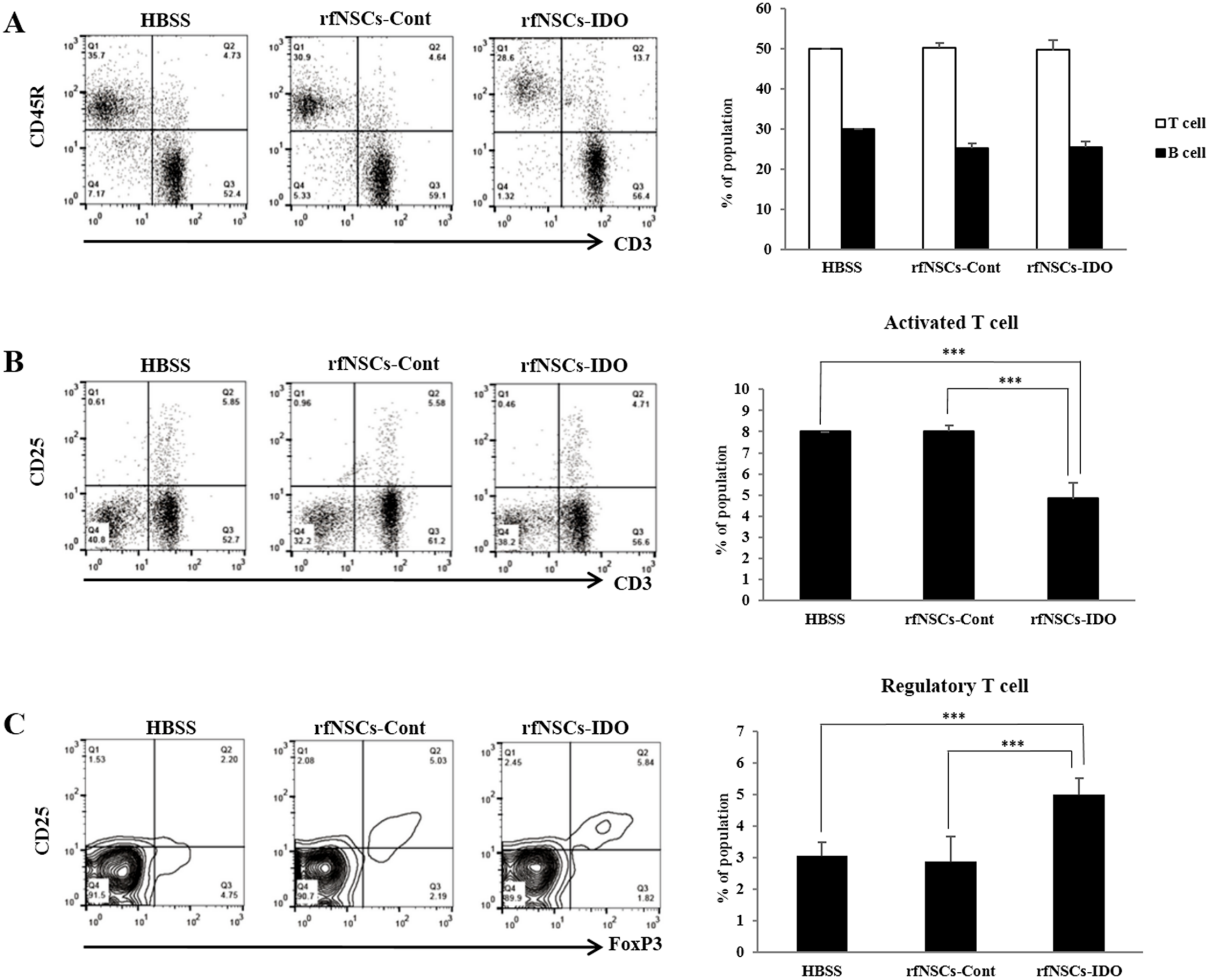
Regulated proliferation of lymphocytes by rfNSCs-IDO *in vivo*. Cervical lymph node samples were collected from animals of each group at 12 dpi. The number of T cells, B cells, activated T cells, and regulatory T cells isolated from sacrificed animals was quantitated by FACS analysis. (A) The number of T cells and B cells showed no significant difference among experimental groups. (B) However, the number of activated T cells was reduced in the rfNSCs-IDO group. (C) Moreover, the number of regulatory T cells was significantly increased compared to the other groups (n = 4, *** p < 0.001).

In the analysis of lymphocytes collected from cervical lymph nodes, the ratio of both CD3^+^ T lymphocytes and CD45R^+^ B lymphocytes was not statistically different and this was similar to the results of the spleen lymphocytes (Fig 4A). However, the number of CD25^+^ activated T lymphocytes in the rfNSCs-IDO group showed a significant reduction compared to both the EAE control and the rfNSCs-Cont groups (Fig 4B). Moreover, the number of CD3^+^, CD25^+^, and FoxP3^+^ regulatory T lymphocytes was significantly increased in the rfNSCs-IDO group (Fig 4C). In the case of the spleen, although there was no significant difference among the groups, the rfNSCs-IDO group showed a tendency of decreased number of CD25^+^ activated T lymphocytes compared to the other groups (S1B Fig). Overall, total counts of various lymphocytes and regulatory T cell population from the spleen were not statistically different (S1A and S1C Fig).

These results revealed that the transplantation of either rfNSCs-Cont or rfNSCs-IDO did not contribute to the suppression of systemic inflammatory responses. However, the reduced number of activated T lymphocytes and increased number of regulatory T lymphocytes in the cervical lymph nodes of the rfNSCs-IDO group suggested that transplantation of rfNSCs-IDO could be effective in suppressing the local inflammatory responses.

**Fig 5:**
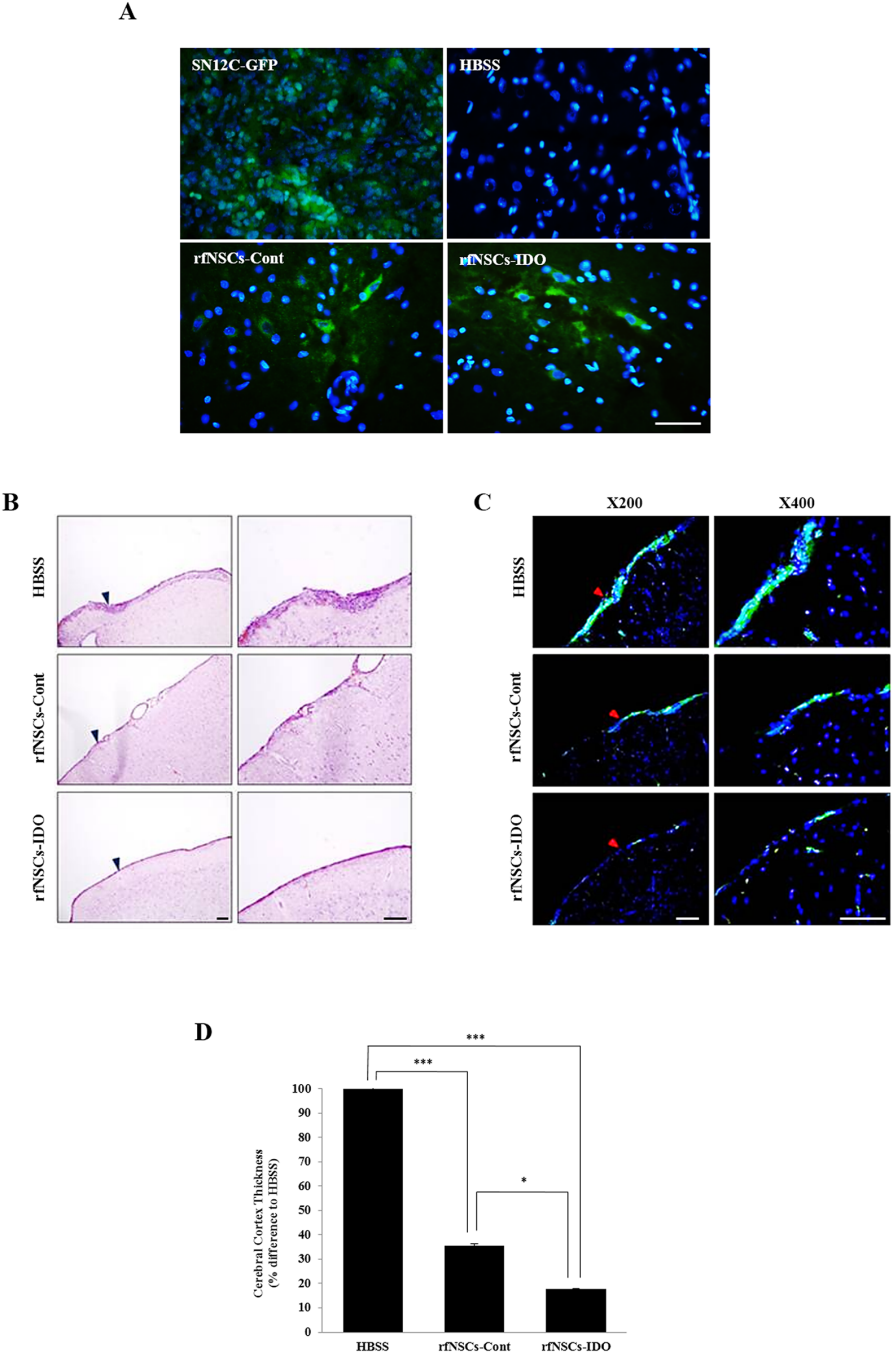
Reduced inflammatory lesion by transplantation of rfNSCs-IDO. The EAE model exhibited the abnormal infiltration of lymphocytes into the brain. (A) Migration of rfNSCs to the cerebral cortex was confirmed by immunofluorescent staining. SN12C-GFP was used as a positive control. Scale bars, 50 μm. (B) Abnormal infiltration of lymphocytes into the cerebral cortex was observed in the HBSS control group, whereas the rfNSCs-Cont and rfNSCs-IDO groups showed less or no lymphocyte infiltration. Blue arrowheads indicated the magnified region of each group. Scale bars, 100 μm. (C) The infiltration of CD25^+^ activated T lymphocyte in the cerebral cortex was visualized by immunofluorescent staining. Red arrowheads indicate the magnified region of each group. Scale bars, 100 μm. (D) The thickness of the lymphocyte layer was measured at the cerebral cortex among the experimental groups. (n = 6, * *p* < 0.05, ** *p* < 0.01).

Next, EAE-induced inflammation in the CNS was visualized and analyzed by immunohistochemistry (IHC). First, we confirmed the migration of rfNSCs to the cerebral cortex by immunofluorescent observation using an anti-EmGFP antibody (Fig 5A). Subcutaneous tumors derived from SN12C renal carcinoma cells expressing EmGFP (SN12C-GFP) were used as positive control for IHC against EmGFP. HBSS injected rat brain tissues were used as negative control. As shown in Fig 5A, we could readily detect rfNSCs-IDO by the expression of EmGFP in the cerebral cortex region. In accordance with the previous reports stating that an abnormal infiltration of lymphocytes following BBB breakage could cause neural damage in the CNS, we found the abnormal infiltration of CD3^+^ T lymphocytes in a broad area of the brain including the cerebral cortex (Fig 5B). Visualized T lymphocytes in cerebral cortex were identified as CD25^+^ activated T lymphocytes (Fig 5C). In comparison to the EAE control group, significantly decreased numbers of CD25^+^ activated T lymphocytes were observed in both the rfNSCs-Cont and the rfNSCs-IDO groups. Moreover, there was a significantly smaller number of activated T lymphocytes visualized in the rfNSCs-IDO group compared to the rfNSCs-Cont group (Fig 5D).

**Fig 6:**
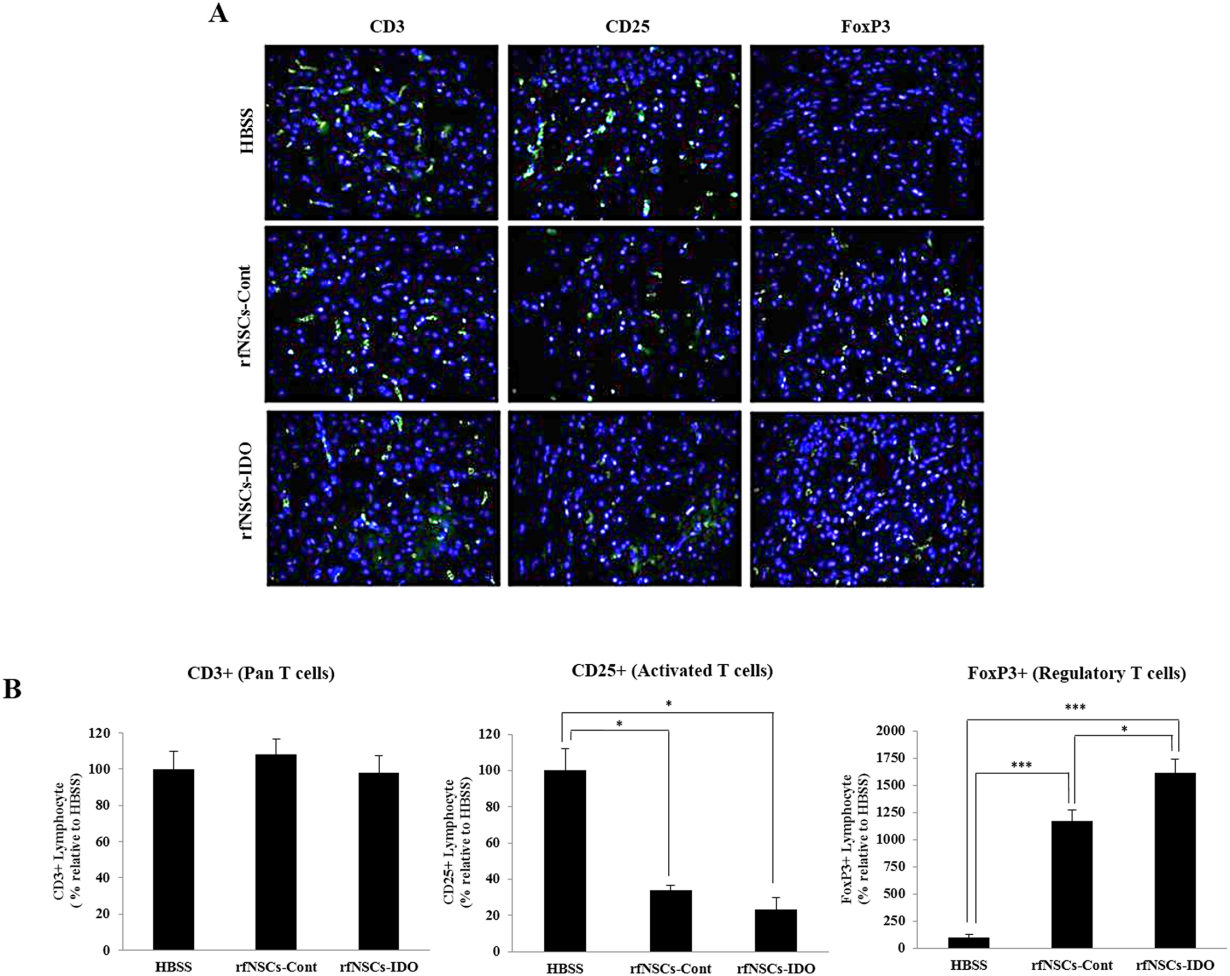
Reduced number of infiltrating activated T lymphocytes by transplantation of rfNSCs-IDO. The EAE model exhibited the abnormal infiltration of lymphocytes into the brain. (A) Immunofluorescent staining was performed against CD3^+^ T lymphocytes, CD25^+^ activated T cells, and FoxP3^+^ regulatory T cells in cerebral cortex. Scale bars, 100 μm. (B) The number of lymphocytes present in the cerebral cortex was quantified. The number of CD3^+^ T cells was not different among experimental groups whereas the number of CD25^+^ activated T cells was significantly reduced in both the rfNSCs-Cont and the rfNSCs-IDO groups compared to the HBSS group. The number of FoxP3^+^ regulatory T cell was greatly increased in the rfNSCs-Cont and the rfNSCs-IDO groups, and the rfNSCs-IDO group showed a significantly increased level compared to the rfNSCs-Cont group (n = 4, * *p* < 0.05, ** *p* < 0.001).

In addition, the cerebral cortical regions were stained against T lymphocytes (CD3^+^), activated T lymphocytes (CD25^+^), and regulatory T lymphocytes (FoxP3^+^). (Fig 6A). In order to compare the number of infiltrating T lymphocytes between the experimental groups, we counted visualized lymphocytes in random sites across the cerebral cortex region (n = 4). Similarly to the FACS results, it was found that the number of CD3^+^ T lymphocytes was not statistically different among experimental groups (Fig 6B). However, compared to the EAE control group, the number of CD25^+^ activated T lymphocytes was significantly reduced by 3- and 5-fold in the rfNSCs-Cont and rfNSCs-IDO groups, respectively (*p* < 0.001). Conversely, the number of FoxP3^+^ regulatory T lymphocytes significantly increased in both the rfNSCs-Cont and rfNSCs-IDO groups. Importantly, the number of activated and regulatory T lymphocytes in the rfNSCs-IDO group was significantly lower and higher than the rfNSCs-Cont group, respectively (Fig 6B).

Taken together, our data suggested that transplantation of rfNSCs played a crucial role in recruiting regulatory T lymphocytes and, in turn, reduced the number of activated T lymphocytes during the inflammation in the CNS. Moreover, transplantation of rfNSCs-IDO could synergistically strengthen the immunosuppressive effects.

## Discussion

The present study describes, for the first time, that the combination of IDO and NSCs could synergistically suppress inflammation by increasing the number of infiltrating immune cells into the brain, which is specifically mediated by CD25^+^ activated T lymphocytes.

NSC is a valuable therapeutic resource against neurodegenerative diseases due to a number of reasons [34–36]. First, because the location of NSCs is a neural microenvironment, after injection into the brain, they migrate and easily engraft into the lesion site of neurodegenerative diseases. Moreover, NSCs possess the potential to regenerate the damaged microenvironment in the CNS by secreting various neurotrophic factors [37,38] and modulating the immune response [39]. Therefore, in response to neural damage, NSC can directly migrate to a lesion site and be easily more adaptable with the neural microenvironment than any other stem cell, which suggests NSCs may be a suitable source for the development of a therapeutic tool.

IDO is an enzyme which was initially discovered from dendritic cells in the pregnant female’s uterus and is known to protect embryo from the host immune rejection [40]. The role of IDO is related with tryptophan metabolism, which drives the recruitment of regulatory T lymphocytes and therefore, modulates the immune response mediated by activated T lymphocytes during inflammation [40,41]. This beneficial effect of IDO has been well studied in mesenchymal cell biology [42,43]. Previous reports stated that mesenchymal stem cells have a strong immune suppressive effect via interferon-γ (IFN- γ) which induces and activates IDO. Conversely, with the inhibited expression of IDO, immune suppression is reversed, which shows the important role of IDO in stem cells on immune suppression [44–47].

Thus, since NSCs do not express IDO themselves, we engineered NSCs to express IDO and examined their synergistically efficacy *in vitro* and *in vivo*. In agreement with previous reports, it was confirmed that NSCs themselves could suppress T lymphocyte proliferation by 2 to 3 fold in the results of MLR. In the case of the rfNSCs-IDO group, T lymphocyte proliferation was effectively suppressed by up to 10 fold at a 1:25 ratio of rfNSCs-IDO:T lymphocytes. An IDO inhibition assay confirmed that immune suppression was mediated by IDO. Interestingly, it was discovered that IDO showed synergistic immune suppression at particular ratios of rfNSCs:T lymphocytes such as 1:25 and 1:50. This result suggested that, in order to actively suppress T lymphocyte proliferation, a minimum concentration of IDO is required in the co-culture with activated T lymphocytes.

Since inflammation and neurodegeneration in the CNS are two major pathological features in MS [48], we used the EAE animal model, mimicking human MS, to validate the activity of rfNSCs-IDO against inflammation in the CNS. Like the *in vitro* immune suppression results, the rfNSCs-IDO group showed greatly improved immune suppression in the CNS. Apparent features of rfNSCs-IDO injected EAE animals included less decrease of body weight and better clinical score, which meant the recovery of clinical conditions following transplantation. In addition, we examined rfNSCs-IDO didn’t affect the total number of T and B cells but had immunosuppresive effect inducing decreased activated T cell and increased regulatory T cell population through FACS analysis. Accordingly, immunohistochemical analysis confirmed that abnormally infiltrating lymphocytes into the brain were reduced in animals after transplantation with rfNSCs-Cont and rfNSCs-IDO. Although the number of total infiltrating T lymphocytes was not different among groups, probably due to the EAE induction prior to rfNSCs transplantation, CD25^+^ activated T lymphocytes were significantly lower in the rfNSCs-Cont and rfNSCs-IDO groups while the number of regulatory T lymphocytes were higher compared to the EAE control group. Importantly, it was found that the number of regulatory T cells was significantly more recruited in the rfNSCs-IDO group than the rfNSCs-Cont group, which suggests that IDO is efficiently functional in the recruitment of regulatory T lymphocytes and might produce neurotrophic factors to recover a damaged microenvironment.

However, it seems to be important to raise several issues which should be examined in the future. Due to current technical limitations of rfNSCs isolation *in vitro*, the cell population may be heterogeneous, consisting of stem cells, progenitor cells, and even differentiated neural populations. Although the majority of heterogeneous cell mixture may be rfNSCs, as we have confirmed, the therapeutic effect on neurodegenerative diseases in the CNS could be maximized by using highly purified rfNSCs. Many studies have reported techniques to obtain a pure NSC population, though there is still controversy in the field [49–51]. Moreover, advanced engineering of NSCs to secrete IDO proteins will be a more effective approach to enhance neurotrophic and paracrine effect in the CNS microenvironment than the current method. Maximizing the chance for IDO to interact with surrounding neural environments is also crucial for improving its therapeutic effect [52,53]. However, it should be considered that not all kynurenines, IDO metabolites, have neuroprotective effects. For instance, kinds of microglial kynurenines (3-hydroxykynurenine, 3-hydroxyanthranilic acid, and quinolinic acid) produce neurotoxic free radicals and exert N-methyl-D-aspartic acid (NMDA) agonist, excitotoxic, activities. [54–56]

In conclusion, it was evident that rfNSCs expressing IDO suppressed inflammation in the CNS. The therapeutic effect of rfNSCs was synergistically increased by the expression of IDO and IDO played a role in the protective mechanism in accordance with known characteristics of rfNSCs. Since inflammation is the one of key features in neurodegenerative diseases, it is necessary to investigate the anti-inflammatory effect of NSCs expressing IDO in other neurodegenerative animal models such as stroke, AD, or PD to further broaden their therapeutic potential.

## Supporting Information

S1 Fig Lymphocytes population analysis in EAE rat spleen. Spleen samples were collected from each group at 12 dpi. The populations of T cells, B cells, activated T cells, and regulatory T cells were quantified by FACS analysis. There were no significant differences among the experimental groups in T cells and B cells (A), activated T cells (B), and regulatory T cells (C) (n = 4).
(TIF)
