# Correction: The Synergistic Local Immunosuppressive Effects of Neural Stem Cells Expressing Indoleamine 2,3-Dioxygenase (IDO) in an Experimental Autoimmune Encephalomyelitis (EAE) Animal Model

**DOI:** 10.1371/journal.pone.0148720

**Published:** 2016-02-03

**Authors:** Young Eun Lee, Jaeyeol An, Kee-Hang Lee, Sung Su Kim, Hye Jin Song, Heejang Pyeon, Hyun Nam, Kyeongjin Kang, Kyeung Min Joo

The following information is missing from the Funding and Acknowledgments sections: This research was supported by a grant (NRF-2013R1A1A2058169) from the National Research Foundation.
