# Supplementary figures and images for: The Synergistic Local Immunosuppressive Effects of Neural Stem Cells Expressing Indoleamine 2,3-Dioxygenase (IDO) in an Experimental Autoimmune Encephalomyelitis (EAE) Animal Model
Source: PLoS One. 2015 Dec 4;10(12):e0144298. doi: 10.1371/journal.pone.0144298 (PMC4670164; doi:10.1371/journal.pone.0144298)

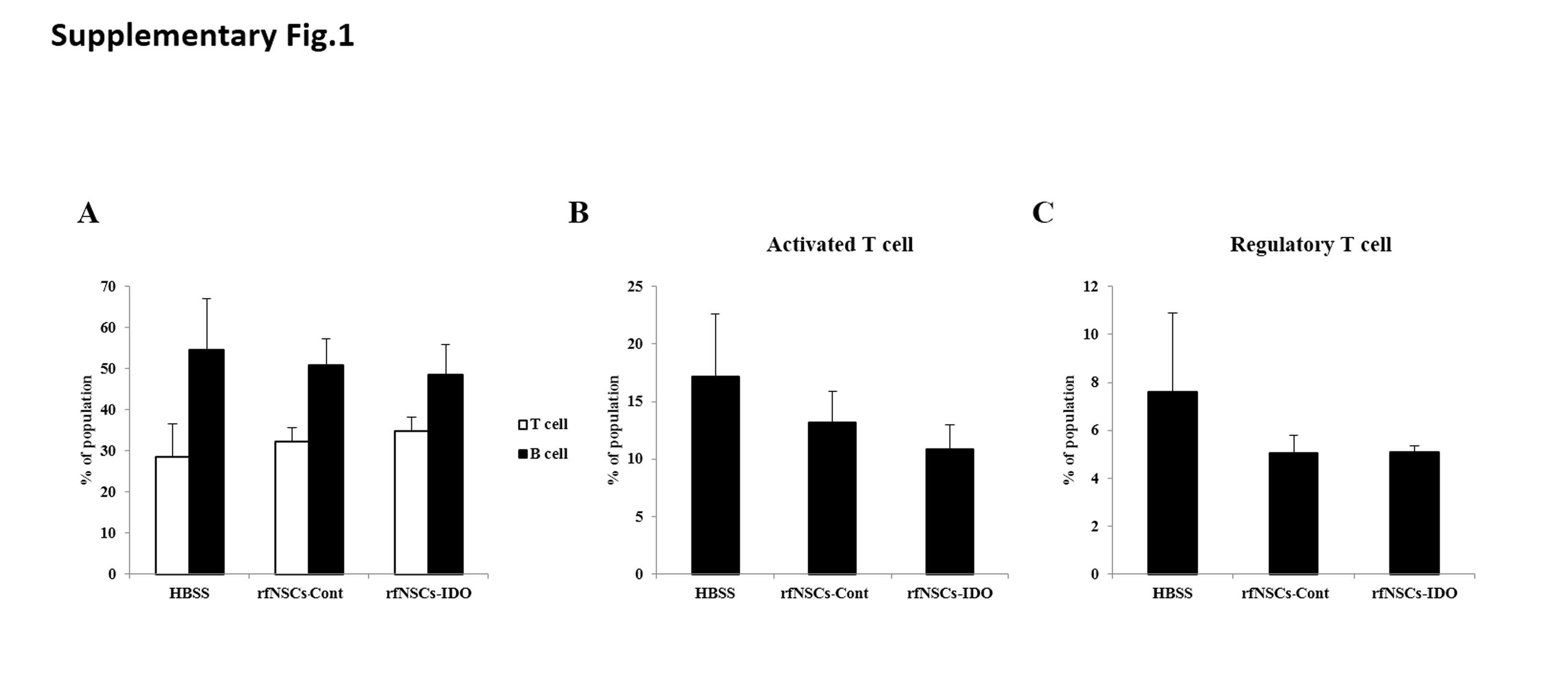

Supplement: S1 Fig — Spleen samples were collected from each group at 12 dpi. The populations of T cells, B cells, activated T cells, and regulatory T cells were quantified by FACS analysis. There were no significant differences among the experimental groups in T cells and B cells (A), activated T cells (B), and regulatory T cells (C) (n = 4). (TIF) [file pone.0144298.s001.tif]
